# Temporal variability of emerging contaminants and estrogenic activity in sediments of a tropical coastal lagoon

**DOI:** 10.1007/s10661-026-15919-5

**Published:** 2026-09-15

**Authors:** Alex da Silva de Freitas, Ana Dalva de Oliveira Santos, Ananda Lima Sanson, Rejany Ferreira dos Santos, Marília Teresa Lima do Nascimento, Giselle Gomes, Allan dos Santos Argolo, Louise da Cruz Félix, Daniele Maia Bila, José Antonio Baptista Neto

**Affiliations:** 1 Instituto de Geociências, Departamento de Geologia, Universidade Federal Fluminense, Niterói, Rio de Janeiro, RJ 24210-346 Brazil; 2 Instituto de Ciências Exatas e Biológicas, Departamento de Química, Universidade Federal de Ouro Preto, Ouro Preto, Minas Gerais, MG 35400-000 Brazil; 3 Fundação Oswaldo Cruz, Cooperação Social, Rio de Janeiro, RJ 21040-900 Brazil; 4 Faculdade de Engenharia, Departamento de Engenharia Sanitária e do Meio Ambiente, Universidade do Estado do Rio de Janeiro, Rio de Janeiro, RJ 20550-013 Brazil

**Keywords:** Endocrine-disrupting compounds, Alkylphenols, Bisphenol A, Estrogenic activity, Yeast estrogen screen, GC–MS

## Abstract

The increasing occurrence of emerging contaminants in aquatic environments has raised concerns about their persistence and ecological impacts, particularly in coastal lagoons subjected to anthropogenic pressure. Here, we investigated the occurrence of pharmaceuticals, alkylphenols, bisphenol A (BPA), and steroid estrogens, together with their estrogenic activity, in surface sediments from Maricá Lagoon (Rio de Janeiro, Brazil), testing the hypothesis that contaminant occurrence and biological responses vary between sampling years despite persistent anthropogenic inputs. Surface sediments were collected from seven fixed sites during dry-season campaigns in 2021 and 2023. Grain size distribution was determined by sediment texture analysis, organic contaminants were identified and quantified using gas chromatography–mass spectrometry (GC–MS), and estrogenic activity was assessed using the yeast estrogen screen (YES) bioassay. Chemical analyses detected alkylphenols (4-octylphenol and 4-nonylphenol), BPA, steroid estrogens, and pharmaceutical-related compounds. BPA was the most frequently detected contaminant (0.10–0.465 ng g⁻^1^), followed by 4-octylphenol (0.010–0.390 ng g⁻^1^) and 4-nonylphenol (0.033–0.344 ng g⁻^1^). Station M7 exhibited the greatest contaminant diversity, with 9 of 12 target compounds detected, identifying it as a localized contamination hotspot. Estrogenic activity was detected in selected samples, whereas pronounced cytotoxicity in one sample may have masked estrogenic responses. Comparisons between campaigns revealed temporal variability in contaminant occurrence and biological responses, although several compounds persisted across both years. These findings demonstrate persistent and spatially heterogeneous contamination and highlight the value of integrating targeted chemical analyses with bioanalytical tools to assess contaminant occurrence and biological effects in coastal ecosystems.

## Introduction

Emerging contaminants (ECs) are increasingly recognized as a significant environmental concern owing to their widespread occurrence, persistence, and capacity to induce adverse biological effects at trace concentrations (Boahen et al., 2025). This diverse group includes pharmaceuticals and personal care products, natural and synthetic hormones, and various industrial chemicals (Zaidi et al., 2025), many of which exhibit endocrine-disrupting properties that interfere with hormonal regulation, reproduction, metabolism, and development in both humans and aquatic organisms (Joshi et al., 2026; Pan et al., 2024). Domestic wastewater and industrial effluents constitute the primary sources of ECs in aquatic environments, although agricultural activities, food processing operations, inadequate solid waste management, and landfill leachates also contribute to their release (Harish & Jegatheesan, 2025; Saini et al., 2026). Owing to their physicochemical properties, many ECs readily adsorb to suspended particles and accumulate in bottom sediments, where they may persist, undergo redistribution, bioaccumulate through aquatic food webs, and act as long-term secondary sources of contamination, particularly in low-energy coastal and lagoonal environments (Chiaia-Hernández et al., 2022; Singh et al., 2025).

Wastewater treatment plants (WWTPs) represent the primary barrier against the release of these pollutants; however, conventional treatment processes are not specifically designed to remove many micropollutants. Consequently, both untreated sewage—particularly in regions with limited sanitation infrastructure—and treated effluents from conventional WWTPs remain important sources of endocrine-active compounds in aquatic environments (Li et al., 2025; Waniek et al., 2025). This issue is particularly relevant in Brazil, where sanitation infrastructure remains insufficient despite ongoing improvements. According to the National Sanitation Information System (SNIS), only approximately 55% of the population has access to sewage treatment services (SNIS, 2020). These limitations are especially evident in the metropolitan region of Rio de Janeiro State, where several municipalities lack comprehensive sewage collection systems or rely on treatment facilities with limited operational efficiency, resulting in the continued discharge of untreated or inadequately treated wastewater into rivers, lagoons, and coastal waters and increasing the vulnerability of these ecosystems to micropollutant contamination (Trata Brasil, 2022).

Tropical coastal lagoons are particularly vulnerable to contaminant accumulation because restricted water exchange, low hydrodynamic energy, and high sedimentation rates promote the retention of particle-associated pollutants (Kebedew et al., 2023; Walton et al., 2024). In addition, these ecosystems often receive diffuse and point-source inputs from densely populated watersheds, making them especially susceptible to the accumulation and long-term persistence of endocrine-active contaminants in sediments (Castaño-Ortiz et al., 2023). Despite increasing recognition of endocrine-active contaminants as a threat to aquatic ecosystems, information on their occurrence and biological activity in tropical coastal lagoon sediments remains limited (Becerra-Rueda et al., 2024; Teixeira et al., 2026). Most studies have focused on rivers, estuaries, or wastewater treatment systems, whereas temporal assessments of sediment contamination in tropical lagoons are still scarce (Ofori et al., 2022; Reyes-Márquez et al., 2022). This knowledge gap limits our understanding of contaminant dynamics and the effectiveness of environmental monitoring in these vulnerable ecosystems.

Against this background, the present study investigated temporal changes in the occurrence of endocrine-active contaminants in surface sediments from Maricá Lagoon, a vulnerable tropical coastal lagoon in southeastern Brazil. We hypothesized that increasing anthropogenic pressures within the watershed have altered the occurrence, concentrations, and spatial distribution of these contaminants between the 2021 and 2023 sampling campaigns. To test this hypothesis, we integrated targeted chemical analyses with the yeast estrogen screen (YES) bioassay to evaluate both contaminant occurrence and sediment estrogenic activity. Specifically, this study aimed to determine (i) which endocrine-active contaminants are present in surface sediments and their concentrations, and (ii) whether contaminant occurrence and estrogenic activity differed between the 2021 and 2023 sampling periods.

## Study area

Maricá Lagoon is part of a system of five interconnected coastal lagoons located in Rio de Janeiro State, southeastern Brazil. Covering an area of approximately 18.74 km^2^ and presenting a mean depth of 1–2 m, the lagoon is characterized by shallow conditions that increase its susceptibility to environmental disturbances and contaminant accumulation (Amorim et al., 2025). Local hydrodynamics are strongly influenced by wind action, which can generate waves reaching heights of up to 1 m, thereby promoting sediment resuspension and the redistribution of particle-associated contaminant. The sedimentary composition varies from sandy to muddy facies, with a predominance of sandy silt and silty sand. These sediments are mainly composed of quartz, feldspar, mica, heavy minerals, shell debris, and lithic fragments (Silvestre et al., 2017). The presence of fine-grained material enhances the capacity for contaminant adsorption, contributing to greater pollutant retention within the system.

Hydrological dynamics within the lagoon complex are primarily controlled by precipitation, as the silting of the Ponta Negra and Cordeirinho channels restricts tidal exchange with adjacent marine waters. This reduced water exchange leads to longer residence times, favoring the accumulation of nutrients and contaminants. Marine water incursions are generally limited and occur predominantly during winter months, when increased wave energy facilitates seawater intrusion (Amorim et al., 2025; Silva et al., 2014).

The lagoon maintains connectivity with the ocean through an intermittent natural inlet located at Barra Lagoon, as well as through a permanent artificial channel (Ponta Negra Canal) constructed in the 1950 s (Amora-Nogueira et al., 2023; Amorim et al., 2025). The regional climate is tropical and controlled by the influence of the South Atlantic Subtropical Anticyclone (SASA) and the periodic passage of cold fronts (Cardoso et al., 2022). These conditions result in elevated temperatures, high atmospheric humidity, and an average annual precipitation of approximately 2000 mm, which collectively drive seasonal water column stratification and variations in hydrodynamic behavior (Silvestre et al., 2015).

Human occupation of the Maricá region has progressively intensified since the colonial period, driving the expansion of coastal settlements and associated economic activities (Mello & Vogel, 2004). The watershed is currently influenced by multiple anthropogenic pressures, including sand extraction, mechanized clay mining, livestock production, and urban expansion (Cruz, 2010). These activities, together with vegetation removal, river channel modification, and the construction of artificial waterways, have increased nutrient and suspended solid inputs to the lagoon system, promoting eutrophication and enhanced sedimentation (Amora-Nogueira et al., 2023; Amorim et al., 2025; Freitas et al., 2023). In combination with limited wastewater treatment capacity and the lagoon’s restricted hydrodynamic connectivity, these factors favor reduced water exchange and the long-term accumulation of contaminants in sediments, increasing the environmental vulnerability of the Maricá Lagoon system.

## Materials and methods

**Fig. 1:**
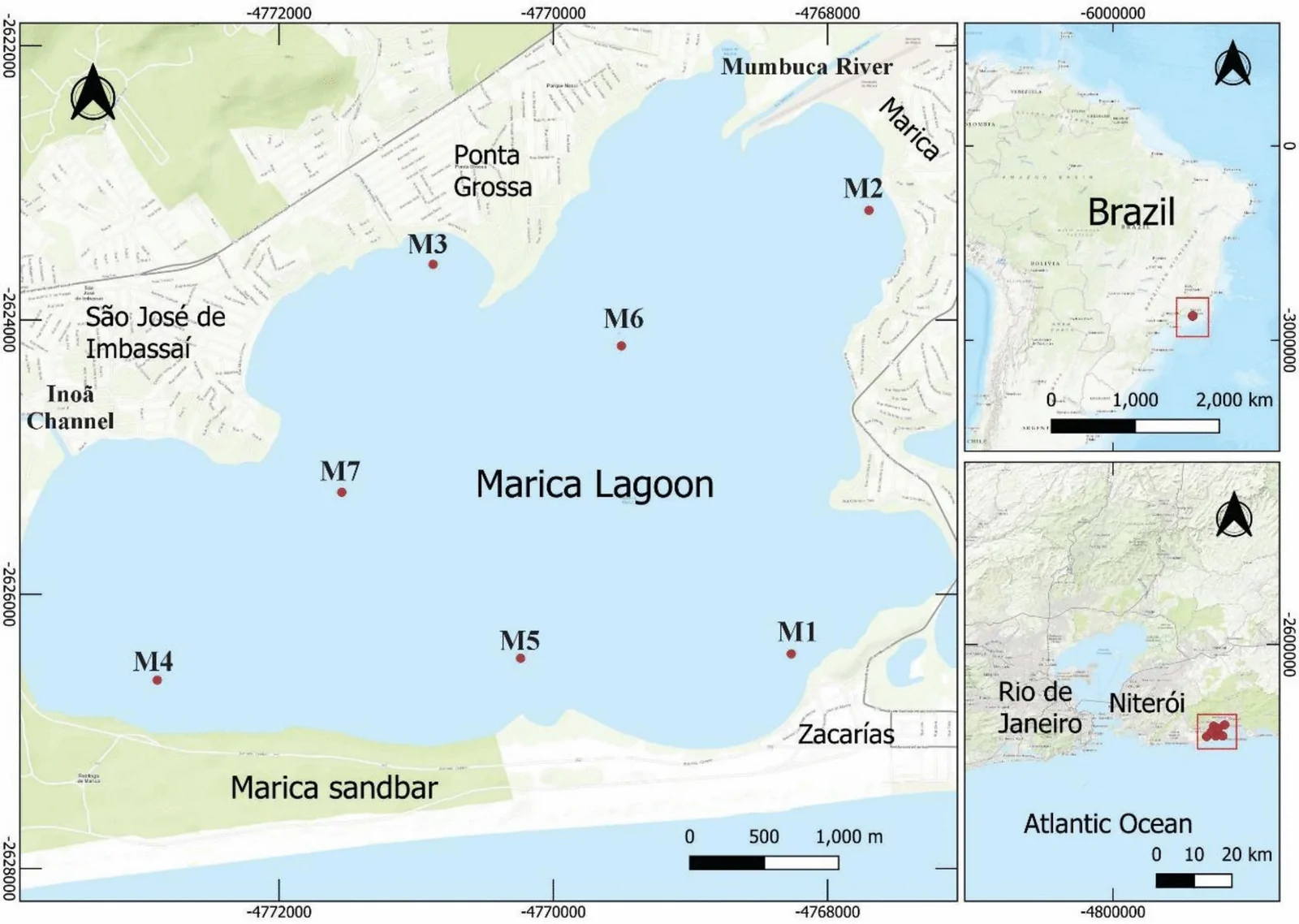
Location of the sampling stations in the Maricá Lagoon, Rio de Janeiro State, Brazil

A total of fourteen surface sediment samples were collected from seven fixed locations during two dry-season campaigns in 2021 and 2023 (*n* = 7 per campaign). Sampling sites were selected to represent the main environmental gradients within Maricá Lagoon, including proximity to known pollution sources (urbanized areas and potential wastewater inputs), zones influenced by marine exchange, and the central basin, as well as previously reported spatial heterogeneity in sediment characteristics (Fig. 1). Surface sediments (0–5 cm) were collected using a Van Veen grab sampler, with one grab per site per campaign. Samples were stored in pre-cleaned glass containers (rinsed with 10% HNO₃ and Milli-Q water), transported under refrigeration, and kept at 4 °C until analysis. The consistent use of fixed sampling locations enabled direct spatial and temporal comparisons between campaigns. Owing to the complexity of the extraction procedures and the need for analytical replication in both chromatographic analyses and the yeast estrogen screen (YES) bioassay, the number of samples analyzed per campaign was restricted to seven. Despite this limitation, the adopted sampling strategy provided a feasible approach for preliminary assessment, enabling the identification of contaminants preserved in sediments.

### Grain size analysis

Prior to analysis, samples were rinsed with distilled water to remove salts. Sediments were analyzed without decarbonation or organic matter removal to preserve their original geochemical composition. Sediment samples were analyzed for grain size distribution to determine textural characteristics, which play a key role in controlling contaminant behavior and accumulation in sediments (Cauwet, 1987). Grain size classification was performed in accordance with the Wentworth (1922) scale. Coarser fractions (sand) were quantified through dry sieving at 0.5 φ intervals, while finer particles (mud fraction) were measured by laser diffraction using a Mastersizer 2000 instrument. Grain size distribution parameters were calculated following the method of Folk and Ward (1957), based on percentile values obtained from the analyses. These parameters were computed using the GRADISTAT software.

### Sample preparation (YES bioassay and GC–MS analysis)

For each sample, 10 g of freeze-dried sediment was placed in glass tubes and extracted with 10 mL of methanol. The mixture was agitated by vortexing for 5 min and subsequently centrifuged at 2500 × g for 5 min to separate the supernatant. This procedure was repeated three times, resulting in a total extract volume of 30 mL. The combined extracts were transferred to a volumetric flask, brought to a final volume of 200 mL with ultrapure water, and acidified to pH 2.0 prior to solid-phase extraction (SPE). The SPE procedure was carried out using an Agilent VacElut 12 manifold fitted with Strata-X cartridges (500 mg, 6 mL). Prior to sample loading, the cartridges were conditioned with 6 mL of hexane, 2 mL of acetone, 6 mL of methanol, and 10 mL of ultrapure water, followed by pH adjustment to 3.0 using 3 mol L⁻^1^ HCl. Samples were then passed through the cartridges under vacuum at a flow rate of 3 mL min⁻^1^. After extraction, the cartridges were rinsed with 10 mL of a methanol–water solution (1:9, v/v) and dried under vacuum using a solid-phase extraction manifold for 10 min. Target compounds were subsequently eluted with 4 mL of acetone, collected in 10 mL glass vials, and evaporated under vacuum. The final residues were reconstituted in 500 µL of acetonitrile for chromatographic analysis (Gomes et al., 2023; Sanson et al., 2014; Santos et al., 2022). The final residue was divided into two aliquots for GC–MS analysis and the yeast estrogen screen (YES) bioassay.

### Yeast estrogen screen (YES) bioassay

Estrogenic activity in sediment samples from Maricá Lagoon was assessed using the yeast estrogen screen (YES) bioassay. Estrogenicity was quantified based on the colorimetric response of the chromogenic substrate chlorophenol red-β-d-galactopyranoside (CPRG). The assay employs the recombinant yeast strain *Saccharomyces cerevisiae* BJ1991, which contains the human estrogen receptor coupled to a β-galactosidase reporter system. Upon interaction with estrogenic compounds, receptor activation triggers enzyme production, leading to CPRG hydrolysis and the development of a measurable color signal. The assay was conducted in 96-well microplates according to the procedure described by Routledge and Sumpter (1996), with minor modifications proposed by (Gomes et al., 2023). Calibration curves were established using 17β-estradiol (E2) in the concentration range of 1.33–2724 ng L⁻^1^, prepared from a 54.48 μg L⁻^1^ stock solution in ethanol. Ethanol was used as the solvent control, while serial dilutions of E2 served as positive controls in each experimental run. Aliquots of sample extracts (10 µL), prepared in absolute ethanol, were dispensed in duplicate into microplate wells and allowed to evaporate to dryness prior to the addition of 200 µL of assay medium containing the recombinant yeast and CPRG (Gomes et al., 2023).

Microplates were sealed, agitated to ensure homogenization, and incubated at 30 °C for 72 h. Following incubation, absorbance was recorded at 540 nm to quantify color development and at 620 nm to correct for turbidity, using a SpectraMax M3 microplate reader. Dose–response relationships for 17β-estradiol (E2) were generated from corrected absorbance values and fitted using a symmetric logistic model implemented in the Origin 6.0 software. Estrogenic activity was expressed as 17β-estradiol equivalents (E2-EQ), obtained by interpolating corrected absorbance values against the E2 calibration curve and accounting for the concentration factor introduced during solid-phase extraction (SPE). For sediment samples, the mean EC50 value for E2 was 38 ± 10 ng L⁻^1^, and the limit of detection (LOD) was estimated at 0.02 ± 0.01 ng L⁻^1^ according to Argolo et al., (2025a, 2025b). Potential cytotoxic effects were evaluated to identify any inhibitory influence of sample constituents on the growth of *Saccharomyces cerevisiae* (Gomes et al., 2023). Growth inhibition was assessed from reductions in turbidity and quantified by measuring absorbance at 620 nm, following Frische et al. (2009). Dose–response relationships were described using a sigmoidal model (Eq. 1), where *A*₁ and *A*₂ represent the maximum and minimum β-galactosidase induction values (corrected absorbance), *x*₀ is the median effect concentration (EC₅₀), *p* is the slope of the dose–response curve, *x* is the sample concentration (ng L⁻^1^ for estradiol equivalents or g L⁻^1^ for sediment extracts), and *y* is the measured response expressed as corrected absorbance.

1 $$y=\frac{{A}_{1}-{A}_{2}}{1+{({x}_{0}/x)}^{p}}$$

### GC–MS (gas chromatography/mass spectrometry) analysis

All glassware was thoroughly cleaned with Extran® neutral detergent (Merck) prior to use. HPLC-grade solvents and reagents were used throughout, and ultrapure water was produced using a Milli-Q purification system (Millipore). Derivatization was applied to target compounds with polar functional groups and high boiling points to improve chromatographic performance by increasing volatility and thermal stability and reducing adsorption and thermal degradation during analysis (Huang et al., 2011). Derivatization reagents included pentafluorobenzyl derivatives, N,O-Bis(trimethylsilyl)trifluoroacetamide (BSTFA), and N-(tert-butyldimethylsilyl)-N-methyltrifluoroacetamide (MTBSTFA), with trimethylchlorosilane (TMCS) used to enhance reaction efficiency (Huang et al., 2011). For derivatization, 100 μL of resuspended extract or standard solution was transferred to a vial with an insert and evaporated under a gentle nitrogen stream. The residue was reconstituted with 25 μL of pyridine containing 200 ppb of PI (internal standard), followed by addition of 75 μL of BSTFA containing 1% TMCS.

The vials were manually shaken and incubated for 30 min at 80 °C, followed by GC–MS analysis. Separation was performed using a ZB-5HT Inferno column (5% phenyl–95% dimethyl polysiloxane; 30 m × 0.25 mm × 0.10 μm) coupled to a Shimadzu QP2010S Plus GC–MS system (Shimadzu®, Brazil) equipped with an autosampler and electronic flow control. Method selectivity was based on retention times and characteristic ion ratios. Instrumental conditions were optimized using a derivatized standard solution (200 μg L⁻^1^) to achieve adequate chromatographic resolution. An initial temperature program was established and subsequently adjusted to reduce run time while maintaining separation efficiency for the 11 target analytes. Compound identification was based on comparison of retention times and mass spectra with the NIST Mass Spectral Library (version 2.0d). Analytes were considered positively identified when ion ratio deviations were within ± 30% of those observed in the reference standard. The optimized method for determining microcontaminants was validated following in-house and inter-laboratory protocols and according to Thompson et al. (2002).

Two calibration curves were prepared by injecting standard solutions at approximately 500 μg L⁻^1^ (ppb) and 50 mg L⁻^1^ (ppm), with concentrations determined by varying the injection volume. Analyses were performed using a Novapak PAH column (4.6 × 250 mm, 5 μm), maintained at 30 °C in a column oven. An injection volume of 20 μL was used, with three replicate injections per sample, and the autosampler was maintained at 18 °C to minimize analyte degradation during analysis. Prior to injection, samples were filtered using syringes equipped with Millex® GV polyvinylidene fluoride (PVDF) membrane filters (0.22 μm pore size). For bisphenol A (BPA), method validation was performed according to the INMETRO (2010) guidelines for analytical methods, including the evaluation of linearity, limits of detection (LOD), limits of quantification (LOQ), and recovery. Linearity was assessed from calibration curves using the correlation coefficient (*R*^2^). The LOQ was defined as the lowest concentration within the calibration range, whereas the LOD was calculated according to Eq. 2, where SD represents the standard deviation of three injections of the blank sample (Sanson et al., 2014; Santos et al., 2022).

2 $$\mathrm{L}\mathrm{O}\mathrm{D}\hspace{0.17em}=\hspace{0.17em}\mathrm{S}\mathrm{D}\hspace{0.17em}\times \hspace{0.17em}6.965$$

## Results

The results from the two sampling campaigns were compared to assess potential temporal variations in sediment contamination and estrogenic activity across the study area.

### Sediment grain size distribution

**Fig. 2:**
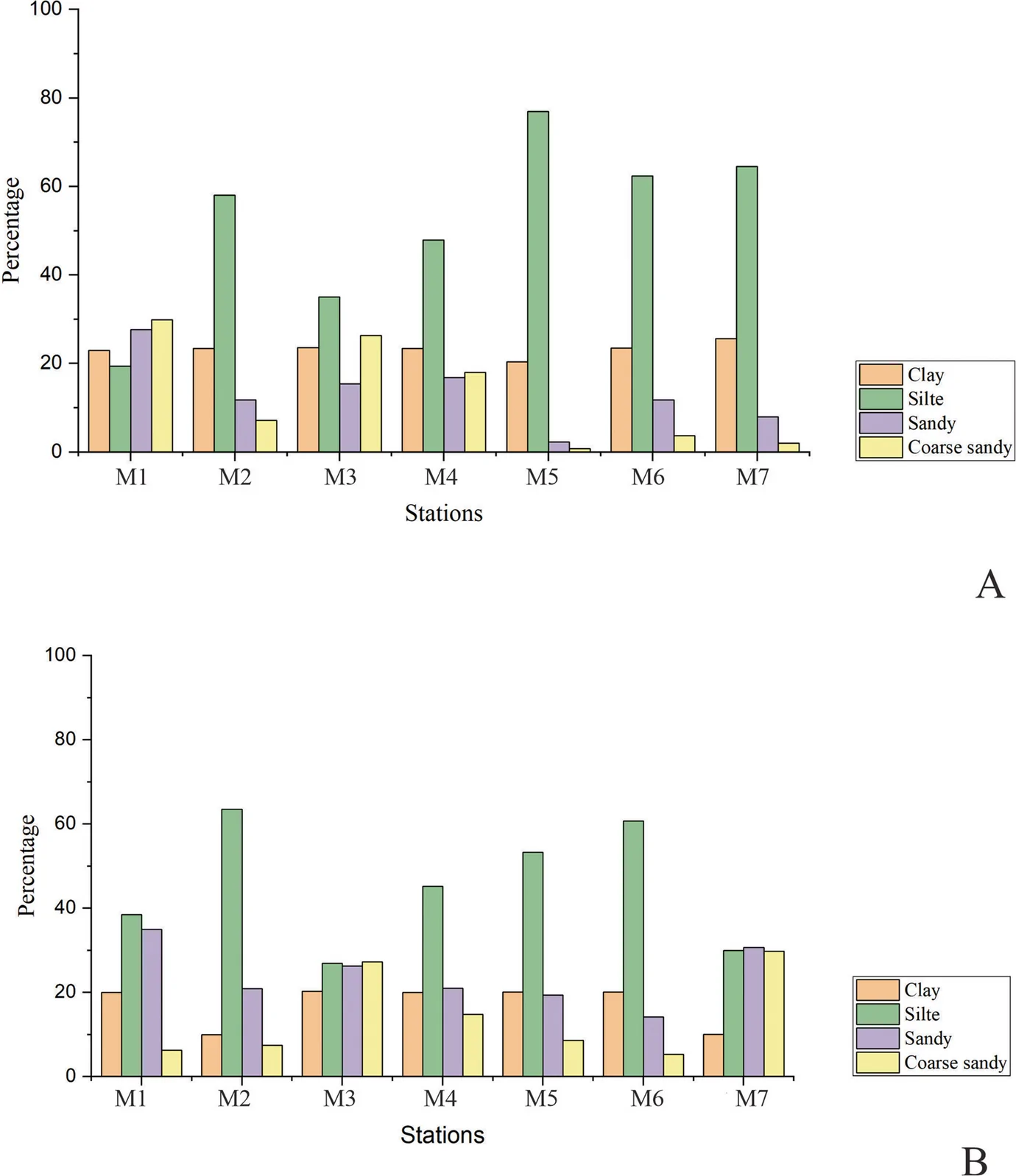
Percentage distribution of sediment grain size fractions in surface sediments from the Maricá Lagoon, Rio de Janeiro State, Brazil, for samples collected in **A** 2021 and **B** 2023

Micropollutants were detected in sediment samples spanning different grain size characteristics, with no consistent pattern between contaminant occurrence and sediment particle size. Grain size analysis revealed a predominance of the silt fraction across most sampling sites in both campaigns. In 2021, sediments were mainly composed of silt, with contributions ranging approximately from 20 to 77%, followed by clay (approximately 20–26%), sand, and coarse sand fractions, which showed greater spatial variability. In 2023, silt remained the dominant fraction at most stations, ranging approximately from 27 to 63%, whereas sand and coarse sand exhibited higher variability among sites. Comparison between sampling campaigns revealed temporal changes in sediment texture, mainly associated with variations in the relative contribution of silt and sandy fractions, with some stations showing shifts of more than 20 percentage points between years (Fig. 2).

### Estrogenic activity in sediment samples

**Fig. 3:**
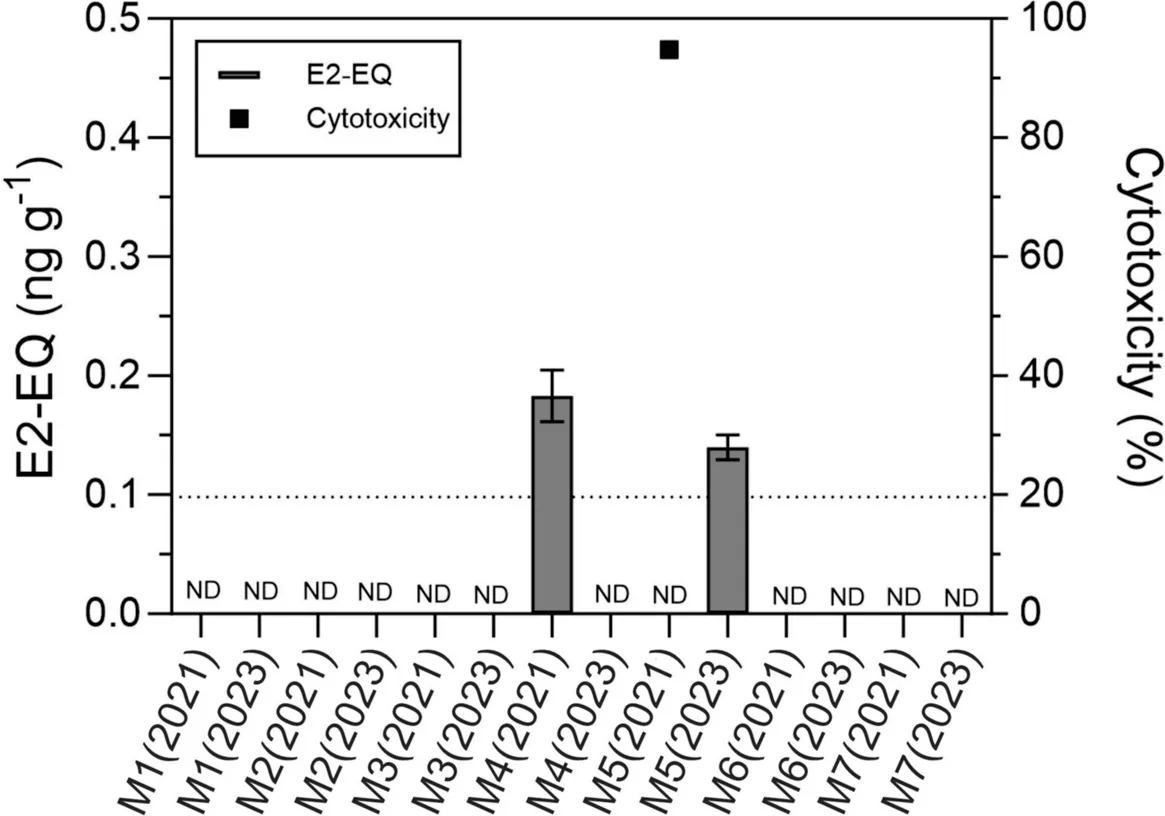
Estrogenic activity (ng g⁻^1^) and cytotoxicity (%) detected in samples collected at the sampling stations of the Maricá Lagoon, Rio de Janeiro State, Brazil

Estrogenic activity ranged from 0.01 to 0.18 ng E2-EQ g^−1^, expressed as 17β-estradiol equivalents, was detected by the yeast estrogen screen (YES) bioassay in samples collected at stations M4 in 2021 and M5 in 2023. Marked cytotoxicity was observed in sample M5 (2021), with cell viability reduced to 5% (95% cytotoxicity). No detectable estrogenic activity (E2-EQ < 0,1 ng g^−1^) was observed in the remaining samples. Based on these bioassay results, targeted chromatographic analyses were subsequently performed to characterize the chemical composition of the samples and to identify compounds potentially contributing to the observed endocrine activity (Fig. 3).

### Micropollutant distribution

Chromatographic analysis detected 9 of the 12 target compounds across the two sampling campaigns, including phenolic compounds, pharmaceuticals, steroid hormones, and bisphenol A (BPA). Overall, BPA was the most frequently detected compound, occurring in nearly all samples and exhibiting a marked increase in concentration between 2021 and 2023, from 0.10 to 0.99 ng g⁻^1^. A similar increasing trend was observed for alkylphenols, with 4-octylphenol ranging from 0.010 to 0.390 ng g⁻^1^ and 4-nonylphenol from 0.033 to 0.344 ng g⁻^1^, indicating sustained or increasing inputs over time. Spatially, steroid hormones were detected at a limited number of sites in 2021, with estrone occurring at M5 (0.128 ng g⁻^1^) and M6 (0.052 ng g⁻^1^), while in 2023 it was detected only at M6 (0.151 ng g⁻^1^), suggesting spatially restricted but persistent contamination at this location. In contrast, station M7 in 2023 showed the highest contaminant diversity, with simultaneous detection of all monitored steroid hormones (estrone, 17β-estradiol, ethinylestradiol, and estriol), as well as pharmaceuticals (gemfibrozil, 0.375 ng g⁻^1^; diclofenac, 0.30 ng g⁻^1^) and alkylphenols, indicating a potential hotspot of mixed contamination within the lagoon system (Table 1). Overall, results indicate both temporal increases in key endocrine-disrupting compounds and strong spatial heterogeneity, with localized hotspots of multi-class contamination.

**Table 1:**
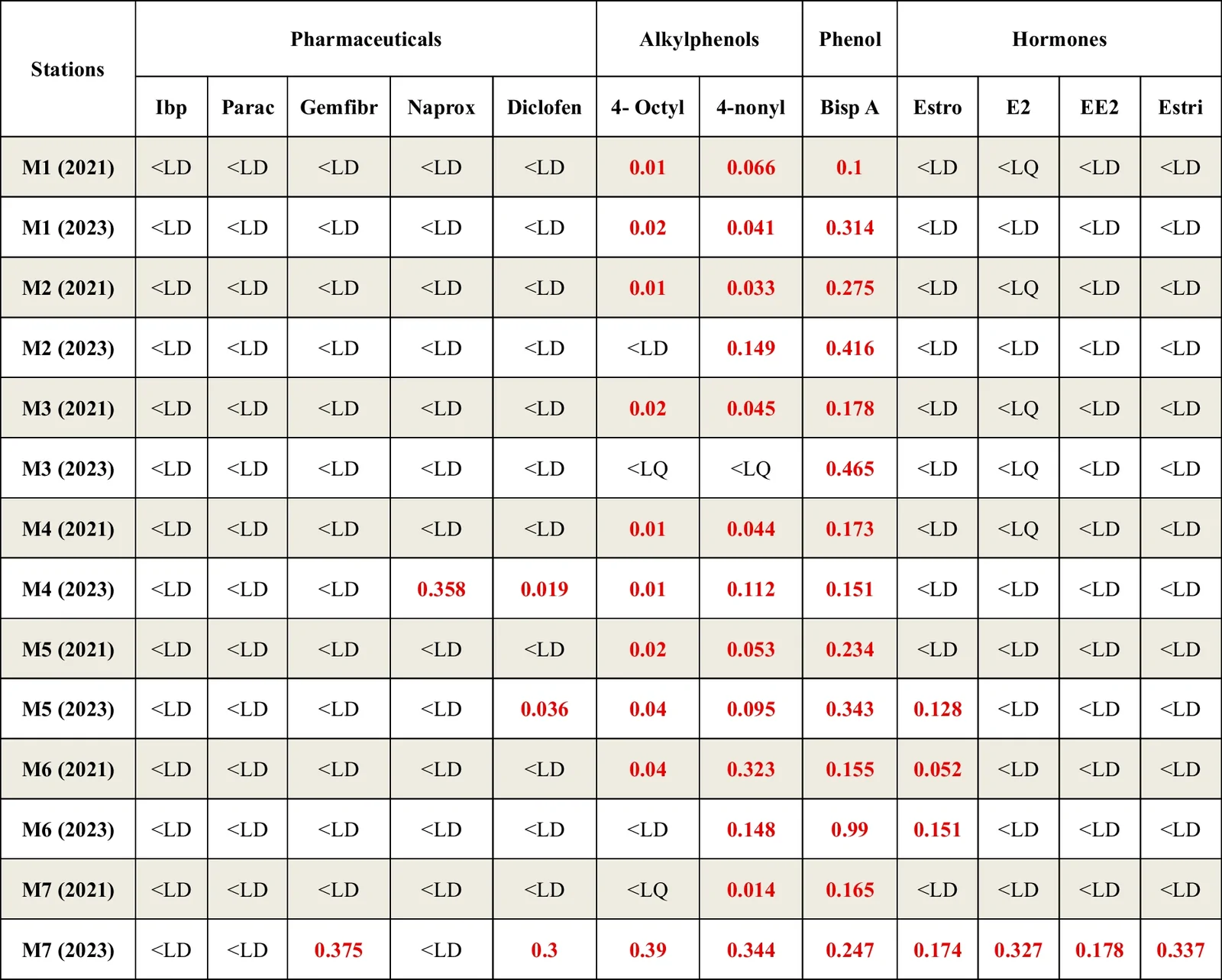
Concentrations (ng g⁻^1^) of micropollutants identified in surface sediment samples from Maricá Lagoon, southeastern Brazil. *Ibp*, ibuprofen; *Parac*, paracetamol; *4-octil*, 4-octylphenol; *4-nonyll*, 4-nonylphenol; *Gemfibr*, gemfibrozil; *Naprox*, naproxeno; *BPA*, bisphenol A; *Diclofen*, diclofenac; *Estro*, estrone; *E2*, 17β-estradiol; *EE2*, 17α-etinilestradiol; *Estri*, estriol

The coloured entries indicate the values detected in the analysed samples by GC–MS

## Discussion

This study demonstrates the occurrence of endocrine-active contaminants in sediments from Maricá Lagoon, with alkylphenols, bisphenol A (BPA), steroid estrogens, and pharmaceuticals detected across both sampling campaigns. BPA and alkylphenols were the most consistently detected compounds, indicating persistent inputs and environmental persistence, consistent with their widespread use and continuous release through wastewater discharges (Corrales et al., 2015; Soares et al., 2008; Ying et al., 2002). In contrast, steroid estrogens and pharmaceuticals exhibited more spatially restricted distributions, with the greatest compound diversity observed at station M7, identifying this site as a localized contamination hotspot. The yeast estrogen screen (YES) bioassay is widely used as a robust approach for assessing estrogenic activity in environmental samples and consumer products (Colosi & Kney, 2011; Routledge & Sumpter, 1996; Soto et al., 1995). Due to its operational simplicity, sensitivity, and cost-effectiveness, the YES bioassay has been broadly applied to screen for endocrine-disrupting compounds in wastewater, surface waters, sediments, and complex environmental mixtures (Bergmann et al., 2020; Gomes et al., 2023; Leusch et al., 2010). Despite these advantages, certain limitations should be considered when analyzing complex environmental matrices. Such samples often contain multiple compounds that may undergo chemical or biological transformations, producing metabolites that differ from the original substances (Ramirez et al., 2014).

These transformations can modify the overall estrogenic activity of the mixture, potentially resulting in underestimation or false-negative outcomes. Furthermore, the presence of highly cytotoxic substances may inhibit yeast growth or interfere with reporter gene expression, thereby masking both estrogenic responses and cytotoxic effects (Gomes et al., 2023). Estrogenic activity measured by the yeast estrogen screen (YES) bioassay was detected only in a subset of samples, while cytotoxic effects were observed in one case. The observed mismatch between chemical detection and estrogenic activity likely reflects the combined influence of bioavailability constraints, mixture interactions, and bioassay-specific limitations. Although several estrogenic compounds, including steroid hormones and alkylphenols, were detected in sediment extracts, their effective bioavailability to the yeast system may have been reduced by strong sorption to sediment organic matter or co-extracted matrix components, limiting receptor accessibility.

In addition, environmental mixtures may contain compounds with antagonistic or inhibitory effects that modulate or suppress estrogen receptor activation, resulting in non-additive biological responses. Such interactions are common in complex environmental matrices, where co-occurring contaminants can interfere with ligand–receptor binding or downstream transcriptional activity, thereby altering the expected estrogenic signal (Argolo et al., 2023; Petrovic et al., 2004). Finally, cytotoxic effects observed in specific samples may have further reduced assay sensitivity by inhibiting yeast growth or compromising reporter gene expression, effectively masking estrogenic responses even in the presence of active compounds. Together, these factors highlight that chemical presence alone does not necessarily translate into measurable biological activity, particularly in complex sediment matrices where mixture effects and matrix interference play a critical role in shaping observed endocrine responses.

Complex environmental mixtures may also contain compounds capable of inhibiting yeast growth or interfering with receptor-mediated responses, which can obscure estrogenic signals and result in false-negative outcomes (Frische et al., 2009; Gomes et al., 2023; Ramirez et al., 2014). The marked cytotoxicity observed in sample M5 (2021) may have contributed to this effect, as elevated toxicity can suppress yeast proliferation and disrupt receptor-mediated signaling pathways. These observations underscore the importance of combining chemical analyses with bioassays to achieve a more comprehensive evaluation of contaminant occurrence and associated biological effects in environmental samples. The integration of bioanalytical approaches with targeted chemical identification therefore represents a more reliable strategy for assessing endocrine-disrupting compounds in complex matrices. Interestingly, no estrogenic activity was detected in this sample, despite it being the only one in which both 17β-estradiol (E2) and ethinylestradiol (EE2)—among the most potent estrogenic compounds—were quantified. The detection of E2 at concentrations exceeding those of estrone (E1) is atypical, given that E1 is generally more prevalent in environmental systems. Estrone is commonly produced as a metabolite of E2 during biological excretion, and subsequent microbial processes further facilitate this transformation (Bai et al., 2025; Combalbert & Hernandez-Raquet, 2010). This apparent inconsistency may reflect limitations in the sensitivity of the YES bioassay, reinforcing the need for complementary analytical techniques to provide a more complete characterization of complex environmental samples.

The widespread occurrence of BPA and alkylphenols across both campaigns suggests continuous anthropogenic inputs, most likely associated with wastewater discharges, consistent with their known environmental persistence and endocrine-disrupting properties (Corrales et al., 2015; Klančič et al., 2022; Newsted et al., 2023). Their accumulation in sediments reflects their affinity for particulate phases and long-term stability in depositional environments (Chiaia-Hernández et al., 2022). Steroid estrogens and pharmaceuticals were primarily associated with station M7, which also exhibited the highest compound diversity. This spatial pattern suggests localized inputs of mixed urban wastewater origin. The detection of diclofenac across both sampling years indicates persistence or sustained inputs, while the presence of gemfibrozil and naproxen only in 2023 suggests temporal variability in contaminant loading or transport processes (Feo et al., 2020; Silva et al., 2011). These compounds have also been linked to estrogenic responses in bioassays, including the yeast estrogen screen (YES), underscoring their relevance as contributors to endocrine disruption in aquatic organisms. Their consistent detection in the present study indicates ongoing anthropogenic inputs, most likely related to wastewater-derived sources. Furthermore, some of the identified compounds, particularly alkylphenols and bisphenol A (BPA), have been shown to interact with estrogen receptors and induce estrogenic responses in vitro. These compounds may therefore contribute to the estrogenic activity observed in environmental samples, although the measured biological response likely reflects the combined effects of multiple contaminants present in the sediment matrix (Ji et al., 2019; Roledo et al., 2024).

Estriol (E3) is a naturally occurring hormone excreted in human urine, with concentrations varying according to physiological conditions such as menstruation, menopause, and pregnancy (Jin et al., 2025). In contrast, E2 and EE2 are widely recognized for their strong endocrine-disrupting potential and are frequently reported as emerging contaminants in aquatic systems (Latosińska & Grdulska, 2025; López-Velázquez et al., 2025). The synthetic estrogen EE2, commonly used in oral contraceptives, is known to exhibit higher estrogenic potency and toxicity compared to natural estrogens such as E1 and E2 (Hashem et al., 2025). Its environmental behavior may also be affected by interactions with co-occurring contaminants, including bisphenol A (Dogra et al., 2025). The presence of these steroid estrogens reinforces the role of sediments as reservoirs for endocrine-disrupting compounds within aquatic environments. As integral components of aquatic systems, sediments serve as important sinks for a wide range of pollutants (Chiaia-Hernández et al., 2022). However, the occurrence and spatial distribution of micropollutants are influenced by a combination of biotic and abiotic factors (Karthik & Dey, 2025). In the present study, sediment grain size alone did not fully explain contaminant distribution, as micropollutants were detected across samples with differing particle-size characteristics. This observation suggests that additional processes, such as hydrodynamic transport and sediment resuspension, likely play a significant role in controlling pollutant dispersion within the lagoon system. Sediment characteristics alone did not explain contaminant distribution patterns, indicating that additional physical processes regulate contaminant fate in the lagoon. Although fine-grained sediments typically enhance sorption of hydrophobic contaminants, hydrodynamic forcing appears to play a dominant role in redistributing contaminants across the system. Wind-driven resuspension associated with regional atmospheric circulation and water exchange driven by riverine inflows and channel connectivity likely contribute to contaminant transport and the formation of localized accumulation zones (Cardoso et al., 2022). These processes are particularly relevant in semi-enclosed coastal lagoons, where restricted water exchange enhances sediment–water interactions and contaminant retention (Burton & Johnston, 2010; Zoumis et al., 2001).

Furthermore, water circulation driven by inflows from the Mumbuca River and the Inoã Channel—both recognized as sources of anthropogenic inputs—likely contributes to the redistribution of contaminants across the system. The environmental risk posed by these compounds is therefore closely linked to their potential remobilization from bottom sediments and subsequent uptake by aquatic organisms (Burton & Johnston, 2010; Zoumis et al., 2001). Among the five pharmaceutical compounds investigated in this study, only three were detected in sediment samples. To the best of our knowledge, this represents the first report of these substances in surface sediments from Maricá Lagoon. While previous studies have documented the occurrence of PAHS, OCs, PCBs, PBDEs, BPA and pesticides in this environment (Gil et al., 2026), investigations specifically addressing pharmaceutical residues remain limited. Pharmaceuticals comprise a diverse group of compounds designed for the prevention and treatment of diseases, and their widespread use has led to increasing detection in aquatic environments. Their presence in surface and groundwater has emerged as a global environmental concern (Khetan & Collins, 2007). These substances can enter aquatic systems through various pathways, including treated and untreated wastewater discharges, hospital effluents, releases from pharmaceutical manufacturing, and improper disposal practices. As conventional wastewater treatment processes are not specifically optimized for their removal, pharmaceutical residues may persist and be transported to receiving water bodies, where they can accumulate in sediments and pose ecological risks (Gaw et al., 2014).

Even at low concentrations, chronic exposure to mixtures of endocrine-active compounds and pharmaceuticals may adversely affect reproductive, developmental, and physiological functions in aquatic organisms, particularly in benthic species in prolonged contact with contaminated sediments (Becerril Ortiz & Ramírez García, 2025; Falfushynska et al., 2025; Hernández-Zamora et al., 2025). Pharmaceuticals detected in 2021 and 2023 indicate persistence within the lagoon system, with diclofenac showing recurrent occurrence across both campaigns, suggesting either continuous inputs or long-term stability in sedimentary compartments. In contrast, gemfibrozil and naproxen were detected only in 2023, which may reflect temporal variability in contamination sources or changes in hydrological and depositional conditions controlling their transport and accumulation. Pharmaceuticals in aquatic environments are commonly associated with wastewater treatment plant (WWTP) effluents, which represent a major pathway for their entry into receiving waters. Their distribution may also be influenced by hydrological processes, including riverine inputs, particularly in regions with limited or inefficient wastewater treatment infrastructure (Feo et al., 2020; Silva et al., 2011). Although present at low concentrations, these compounds may still contribute to ecological risk in aquatic systems (Becerril Ortiz & Ramírez García, 2025; Falfushynska et al., 2025; Hernández-Zamora et al., 2025). Similar occurrence patterns have been widely reported in sediments globally, with variability driven by wastewater inputs, hydrodynamics, and treatment efficiency (Feo et al., 2020). Overall, the presence of diclofenac and other pharmaceuticals in Maricá Lagoon is consistent with contamination patterns observed in urban-impacted coastal systems (Becerra-Rueda et al., 2024; Castaño-Ortiz et al., 2023).

The comparison between the 2021 and 2023 sampling campaigns indicates temporal variability in both contaminant occurrence and associated biological responses, reflecting dynamic but persistent anthropogenic pressure within the lagoon system. While several compounds, particularly bisphenol A and alkylphenols, were consistently detected across both campaigns, their persistence suggests continuous inputs rather than episodic contamination events. In contrast, the detection of specific pharmaceuticals only in 2023 points to changes in either emission patterns or environmental transport and deposition processes, potentially influenced by hydrological variability or fluctuations in wastewater discharge intensity. The occurrence of steroid estrogens and higher compound diversity at selected sites further supports the presence of temporally and spatially heterogeneous inputs linked to localized anthropogenic sources. Despite these differences in chemical occurrence, the overall persistence of endocrine-active compounds across both campaigns suggests limited system recovery over time. However, the relatively low number of samples exhibiting estrogenic activity in the YES bioassay in both years indicates that temporal changes in chemical presence were not always directly reflected in biological responses, likely due to mixture effects and site-specific variability in exposure conditions. Overall, the temporal patterns observed here highlight the importance of repeated monitoring in dynamic coastal lagoon systems, where contaminant inputs, hydrodynamics, and sediment processes interact to produce non-linear and spatially heterogeneous exposure profiles.

## Conclusions

This study demonstrates the occurrence of endocrine-disrupting compounds and other emerging contaminants in surface sediments from Maricá Lagoon across two sampling campaigns conducted in 2021 and 2023. Alkylphenols, bisphenol A, steroid estrogens, and pharmaceutical-related compounds were detected at multiple sampling sites, with station M7 exhibiting the greatest contaminant diversity, identifying it as a localized contamination hotspot. Comparison of the two sampling campaigns revealed temporal variability in both contaminant occurrence and biological responses, including differences in estrogenic activity and cytotoxicity, highlighting the dynamic nature of contaminant distribution in the lagoon. Nevertheless, the recurrent detection of several contaminants across both campaigns indicates their persistent presence in the sedimentary environment. The combined application of chromatographic analyses and the yeast estrogen screen (YES) bioassay provided complementary information on contaminant occurrence and potential estrogenic effects. However, the observed discrepancies between chemical detection and biological responses underscore the limitations of relying on either approach alone. These inconsistencies likely reflect the influence of bioavailability, contaminant mixtures, unidentified bioactive compounds, and interactions among chemicals, which can modulate biological responses beyond those predicted from targeted chemical analyses. Therefore, integrating chemical and effect-based methods offers a more comprehensive framework for sediment quality assessment while also highlighting the need for broader analytical strategies and complementary bioassays to improve environmental risk characterization. This study provides a baseline for long-term monitoring of emerging contaminants in Maricá Lagoon and highlights the value of integrating chemical and bioanalytical approaches to support coastal management, prioritize monitoring of contamination hotspots, and guide actions to protect lagoon ecosystem health.

## Data Availability

No datasets were generated or analysed during the current study.
